# Mapping Global Trends in *Dirofilaria immitis* Research Within the One Health Framework (1945–2025): A Bibliometric Perspective

**DOI:** 10.3390/ani16060988

**Published:** 2026-03-22

**Authors:** Raúl Aguilar-Elena, Iván Rodríguez-Escolar, Manuel Collado-Cuadrado, Elena Infante González-Mohino, Alfonso Balmori-de la Puente, Alberto Gil-Abad, Rodrigo Morchón

**Affiliations:** 1 Research Group on Occupational Risk Prevention and Occupational Health and Safety (GPRL), PREVION Chair of Occupational Risk Prevention, Valencian International University (VIU), 46002 Valencia, Spain; 2 Zoonotic Diseases and One Health Group, Faculty of Pharmacy, Centre for Environmental Studies and Rural Dynamization (CEADIR), University of Salamanca, 37007 Salamanca, Spain; ivanrodriguez@usal.es (I.R.-E.); manuelcollado@usal.es (M.C.-C.); elena.igm4@usal.es (E.I.G.-M.); a.balmori@usal.es (A.B.-d.l.P.); albertogilabad@usal.es (A.G.-A.); 3 Biomedical Research Institute of Salamanca (IBSAL), University of Salamanca, 37007 Salamanca, Spain

**Keywords:** *Dirofilaria immitis*, heartworm disease, bibliometric analysis, One Health, zoonosis

## Abstract

*Dirofilaria immitis* constitutes a significant global veterinary burden and an emerging zoonotic risk. Despite decades of study, the structural evolution of its scientific landscape remains unexplored. This study provides a comprehensive longitudinal analysis of global research on *D. immitis* to evaluate its trajectory, intellectual structure, and conceptual shifts over the last eight decades. A systematic bibliometric analysis was conducted following PRISMA guidelines adapted for bibliometrics. Data were retrieved from Web of Science Core Collection and Scopus, covering the period from 1945 to 2025. After deduplication and manual screening, a final corpus of 3589 documents was analyzed using performance indicators and science mapping techniques to assess growth patterns, geographic leadership, collaboration networks, and thematic evolution. The field exhibits a mature profile with a sustained mean annual growth rate of 2.39%. Production is geographically polarized, with the United States and Italy acting as the primary research hubs, though international collaboration networks are increasingly integrating endemic regions in the Global South. Thematic analysis reveals a profound paradigm shift: while early research (1945–1980) focused on parasite morphology and clinical description, the 21st century is characterized by a multidisciplinary approach dominated by molecular biology, the study of the endosymbiont *Wolbachia*, and the genetic mechanisms of macrocyclic lactone resistance. The intellectual structure is currently organized into distinct but interconnected clusters, linking established clinical pathology with emerging genomic and environmental control strategies. Research on *D. immitis* has evolved from a classical parasitology discipline into a complex biomedical ecosystem aligned with the One Health framework. The persistence of the disease, driven by drug resistance and climate-mediated vector expansion, has catalyzed a transition toward integrative research models. Future control strategies must transcend geographic borders, combining advanced genomic surveillance with ecological modeling to mitigate the impact of this transboundary disease on both animal and human health.

## Simple Summary

Heartworm disease, caused by the parasite *Dirofilaria immitis*, is a major health threat to dogs and a growing concern for human health. Despite decades of study, the evolution of scientific interest in this parasite has never been mapped. This study reviewed 80 years of global research (from 1945 to 2025) by analyzing 3589 scientific documents. We found that heartworm research has grown steadily, primarily led by the United States and Italy, although researchers from heavily affected regions in the Global South are increasingly participating. The focus of these studies has changed dramatically. Early research mainly described the parasite’s appearance and the clinical symptoms it caused. Today, scientists are focused on its genetics, a symbiotic bacterium it carries called *Wolbachia*, and the alarming issue of the parasite becoming resistant to preventative drugs. Ultimately, studying heartworm is no longer just about basic biology; it requires a global “One Health” approach connecting animal, human, and environmental health. Because climate change is helping the mosquitoes that spread the disease expand to new areas, future strategies must combine genetic monitoring with environmental tracking to control this widespread threat.

## 1. Introduction

*Dirofilaria immitis* is a parasitic nematode that causes heartworm disease, a zoonotic condition representing a complex health challenge that exemplifies the need for a One Health approach due to its high sanitary impact on animals and humans across multiple world regions [1,2]. Its primary definitive hosts are domestic and wild canids, with the domestic dog being the species for which the most data are available; however, it can also infect felids and other mammals, including humans as accidental hosts. In recent decades, a geographic expansion and shifts in distribution have been documented, associated with climatic, ecological, and anthropogenic factors [3].

Adult *D. immitis* worms reside in the pulmonary artery and the right ventricle of the heart of their definitive host. The viviparous females produce microfilariae that are released into the bloodstream. Transmission is vector-borne, mediated by culicid mosquitoes of various genera (mainly *Culex* sp., *Aedes* sp., and *Anopheles* sp.). These vectors ingest microfilariae from the host’s peripheral blood, which then develop into infective larvae to be transmitted during subsequent blood meals. Ambient temperature conditions larval development within the mosquito; consequently, only specific thermal windows allow for larval maturation and, therefore, effective transmission [2].

In domestic dogs, *D. immitis* causes potentially severe cardiopulmonary disease, characterized by the development of pulmonary hypertension, endothelial damage, thromboembolism, and, in advanced cases, right-sided heart failure and caval syndrome. Dogs may remain asymptomatic or present with cough, exercise intolerance, weight loss, and signs of heart failure, depending on the parasite burden and the duration of the infection. In cats, infection typically involves a lower number of parasites but can produce acute respiratory symptoms and pulmonary lesions disproportionate to the parasitic load, carrying a risk of sudden death [4]. In humans, however, the infective larvae usually die in the distal pulmonary arterial branches, generating, in some cases, a solitary, calcified peripheral nodule known as human pulmonary dirofilariasis. Most patients are asymptomatic, and the finding is often incidental during imaging tests; however, the nodule can mimic a primary or metastatic neoplasm, leading to unnecessary thoracic surgery if a parasitic etiology is not considered [5].

Various reviews, systematic reviews, and meta-analyses have highlighted that heartworm disease constitutes a major animal health problem on a global scale. Its distribution is modulated by environmental factors (climate, vector habitats), land-use changes, the mobility of animals and humans, and veterinary prevention and diagnostic practices [2,5,6,7]. A global meta-analysis on infection in dogs estimated a weighted global prevalence of *D. immitis* in dogs of 10.91%, confirming the wide distribution and impact of this parasite on the canine population [6]. At the continental level, there are exhaustive analyses in Asia [8,9], Africa [10], Australia [11], Europe [3,4,12,13], and the Americas [2,7,14,15,16,17] that delve into the complexities of this parasite.

However, despite the growing scientific production and the veterinary and zoonotic importance of *D. immitis*, there is to date no specific bibliometric analysis covering the entire historical development of research that systematically explores global research trends on this parasite within a One Health framework. Consequently, the objective of this study was to address the evolution of scientific production and to map and analyze global research trends on *D. immitis* over the last 80 years—since indexed reports have been available—using bibliometric methods. This analysis will provide a macroscopic perspective, identifying international cooperation networks and emerging thematic areas within the One Health framework, offering a roadmap for future research and disease control strategies that are becoming vital in a scenario of climate change and vector expansion.

## 2. Materials and Methods

### 2.1. Study Design

A descriptive and longitudinal bibliometric analysis of the international scientific production related to *D. immitis* was conducted. The aim was to identify the temporal evolution of research, key scientific actors, collaboration patterns, dominant thematic areas, and emerging trends over the period 1945–2025. The methodology was developed following standard recommendations for bibliometric studies published in high-impact journals in the fields of parasitology and public health, ensuring the reproducibility of the process and transparency in data acquisition and processing.

### 2.2. Data Sources and Search Strategy

Scientific literature retrieval was carried out using two international reference bibliographic databases: Web of Science Core Collection (WoS) and Scopus. These databases were selected for their broad multidisciplinary coverage, the quality of their bibliographic records, and their established use in previous bibliometric studies on parasitic and zoonotic diseases.

The search strategy was designed to be parallel and equivalent in both databases, employing the specific scientific name of the species as the primary criterion to maximize thematic precision and avoid the inclusion of marginal literature. The following search queries were used: Scopus: TITLE-ABS-KEY (“*Dirofilaria immitis*”) and WoS: TS = (“*Dirofilaria immitis*”).

Searches were performed without restrictions on language or document type in the initial phase, covering from the inception of available indexing up to and including the year 2025. The exact date of record retrieval was documented to ensure study reproducibility.

### 2.3. Inclusion and Exclusion Criteria

Documents were included if they simultaneously met the following criteria: (1) explicit mention of *D. immitis* in the title, abstract, or keywords (Scopus) or in the “Topic” field (WoS); (2) publication indexed in WoS or Scopus within the analyzed time period; and (3) documents with sufficient complete bibliographic information for analysis (authors, year, source, affiliations, keywords, and references). Duplicate records between the two databases, incomplete documents, indexing errors preventing correct integration into the bibliometric analysis, as well as editorial corrections, errata, or non-scientific records were excluded.

### 2.4. Data Processing and Cleaning

Bibliographic records were exported from WoS and Scopus in compatible formats (BibTeX and/or plain text) and subsequently integrated into a single dataset. Information processing, cleaning, and analysis were performed using R software (version 4.4.1; R Foundation for Statistical Computing, Vienna, Austria) and the integrated development environment RStudio (version 2024.09.0+375; Posit Software, PBC, Boston, MA, USA) primarily utilizing the bibliometrix package (version 5.0.1; University of Naples Federico II, Naples, Italy), which is widely validated in bibliometric studies. The procedure included the following: (1) conversion of original files into a unified bibliographic data frame; (2) identification and elimination of duplicates via matching of DOIs, titles, and authors; (3) normalization of author names, institutions, and countries to avoid artificial fragmentation; and (4) homogenization of keywords where necessary (singular/plural forms, spelling variants).

### 2.5. Bibliometric Analysis

The bibliometric analysis was structured into complementary levels to offer a comprehensive view of the evolution, structure, and impact of scientific research on *D. immitis*. First, a descriptive analysis of scientific production was conducted, including the annual evolution of publication volume over the study period, document typology, publication languages, and the identification of the most productive journals in the field. Second, the scientific contribution of authors, institutions, and countries was examined by studying the scientific productivity of authors and countries, analyzing total and average citations as basic bibliometric impact indicators, identifying the most cited sources and authors, and classifying scientific production by the corresponding author’s country to characterize the geographic distribution of research. Third, scientific collaboration patterns were analyzed by differentiating between Single Country Publications (SCPs) and Multiple Country Publications (MCPs), as well as by constructing country-level co-authorship networks to identify general collaboration structures and relevant nodes within the scientific field. The structure of these networks was examined using standard network analysis metrics implemented in the bibliometrix environment, without performing an individualized analysis of specific centrality metrics. Finally, thematic analysis was developed through the study of author keyword frequency and co-occurrence, the identification of thematic clusters representative of the main research domains, and the analysis of the temporal evolution of research foci, allowing for the distinction between consolidated lines and emerging areas within the field. This multi-level approach integrated descriptive, relational, and thematic indicators, providing a robust characterization of global research trends on *D. immitis*, consistent with methodological recommendations established by guidelines such as PRISMA guidelines adapted for bibliometrics recommendations.

Bibliometric visualizations, including collaboration maps, keyword networks, and thematic diagrams, were generated using the functions within the bibliometrix package and complementary visualization tools in R statistical software (v.4.2.3). Author productivity analysis was performed automatically via bibliometrix, without applying manual filters or exclusions related to the authorship of the researchers signing the present study. 

### 2.6. Flow Diagram of the Study Selection Process

The process of identifying, selecting, and including scientific documents was structured using a PRISMA approach adapted for bibliometric studies, as recommended by recent work in scientometrics and public health research [18,19]. This approach allows for a transparent and reproducible description of the various phases of constructing the bibliographic corpus.

In the initial identification phase, all records indexed in the WoS Core Collection and Scopus were retrieved using the previously defined search equations. The search was conducted without restrictions on document type or language to maximize sensitivity and capture the entirety of scientific production related to *D. immitis* from the start of available indexing through 2025. Subsequently, records from both databases were integrated into a single bibliographic set. In this phase, duplicate elimination was undertaken, identified primarily through DOI, document title, and author matching. This step allowed for the refinement of the initial corpus and prevented the overestimation of scientific production. In the screening phase, the remaining records were reviewed to detect potential indexing errors, incomplete documents, or non-scientific entries (such as errata or editorial corrections) that did not contribute relevant information to the bibliometric analysis. Records failing to meet minimum bibliographic quality criteria were excluded. Finally, documents surpassing all previous phases constituted the final set of studies included in the bibliometric analysis. This definitive corpus was used to analyze the temporal evolution of scientific production, collaboration patterns, co-authorship networks, and the thematic evolution of research on *D. immitis*.

## 3. Results

### 3.1. Document Identification and Selection Process

**Figure 1:**
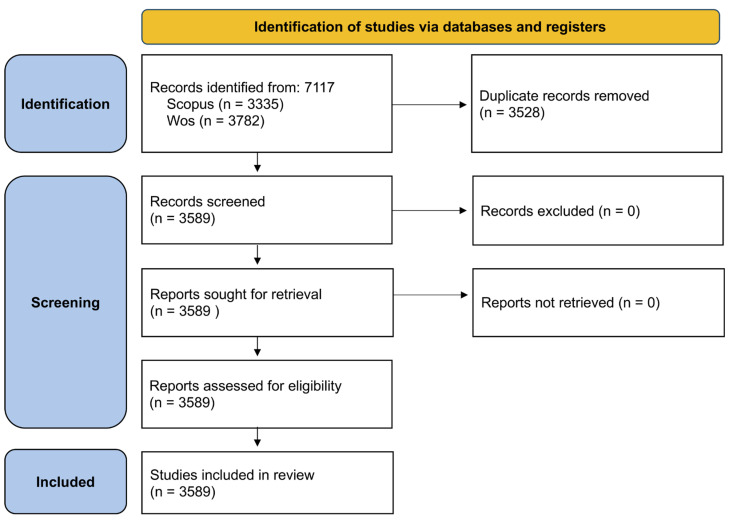
Flow diagram of the identification, screening, and inclusion of records in the bibliometric analysis of *Dirofilaria immitis* research (1945–2025). Records were retrieved from Scopus and Web of Science, duplicates were removed, and the remaining documents were screened and assessed for inclusion in the final bibliometric corpus covering the period 1945–2025.

The document identification and selection process was conducted following a PRISMA approach adapted for bibliometric studies to ensure procedure transparency and reproducibility. In the identification phase, a total of 7117 records were retrieved from the WoS Core Collection (*n* = 3335) and Scopus (*n* = 3782) databases using the previously defined search equations. Following the merger of the databases (WoS and Scopus), records were normalized. Duplicates were removed based on DOI and title matching. Additionally, documents lacking defined authorship or sufficient metadata for bibliometric analysis were manually excluded. This resulted in a final collection of 3589 documents, which were evaluated to verify their bibliographic completeness. Given the highly specific nature of the search strategy employed, no further records required exclusion at this stage. Ultimately, a total of 3589 documents were included in the bibliometric analysis, constituting the definitive corpus of the study. The complete document selection process is summarized in the PRISMA-bibliometric flow diagram. Final data retrieval was performed on 15 January 2026. No language restrictions were applied to avoid publication bias and ensure a global perspective of scientific production within the One Health framework. This bibliometric study was conducted and reported in accordance with the PRISMA guidelines adapted for bibliometrics recommendations, ensuring transparency and reproducibility (Table S1). The bibliometric analysis included a total of 3589 documents published between 1945 and 2025, originating from 628 distinct scientific sources, including specialized journals, conference proceedings, and other academic formats (Figure 1).

The temporal delimitation starting from 1945 allows for a focus on the modern consolidation phase of research on *D. immitis*, excluding isolated historical records with little scientific continuity (Table 1). The mean annual growth rate of scientific production was 2.39%, indicating a sustained increase in research interest over eight decades. The documents have an average age of 19 years, reflecting the coexistence of highly influential classic literature with significant recent production. 

The average citations per document was 18.52, equivalent to 1.29 citations per document per year, suggesting a moderate but stable scientific impact over time. The analyzed corpus included a total of 45,471 bibliographic references, evidencing a broad and cumulative knowledge base. Regarding document typology, original articles constituted the predominant format (*n* = 2638), followed by reviews (*n* = 218), while other document types—notes, editorials, letters, conference papers or short communications—represented a minority proportion. Authorship analysis identified 12,119 authors, with 19,419 author appearances, confirming a markedly collaborative research environment. The average documents per author was 0.30, with a mean of 5.41 co-authors per document; only 281 works were single-authored. International co-authorship appeared in 16.58% of documents, indicating a moderate level of transnational collaboration in the study of *D. immitis*.

### 3.2. Annual Scientific Production

**Figure 2:**
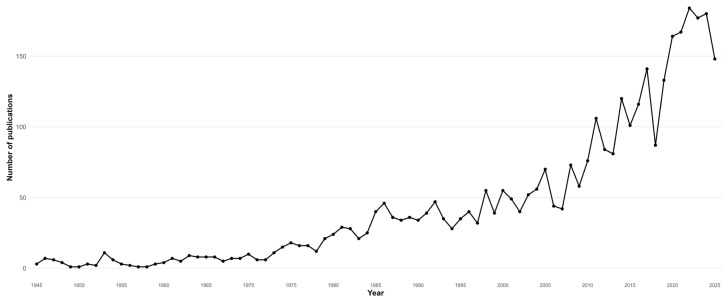
Annual scientific production on *Dirofilaria immitis* research (1945–2025).

The temporal evolution of scientific production on *D. immitis* shows a pattern of progressive growth, with clear differences between historical phases (Figure 2, Table 2). During the 1945–1965 period, annual production was low and relatively irregular, reflecting an initial stage focused mainly on descriptive studies, isolated clinical observations, and basic research. From the late 1960s, and more clearly during the 1970s and 1980s, a gradual increase in publication volume is observed, coinciding with the consolidation of veterinary parasitology and increased interest in vector-borne diseases. Growth intensified from the 1990s onwards, showing a sustained upward trend that continued through the early 21st century. This phase is associated with the incorporation of new diagnostic techniques, advances in molecular biology, and a more systematic approach to the epidemiology of dirofilariasis. In the most recent period (2010–2025), annual production reached its highest values, with sustained peaks in publications per year, reflecting the full consolidation of the field. This increase is linked to both the veterinary relevance of *D. immitis* and its growing interest within the context of public health and the One Health approach.

### 3.3. Most Productive Authors

Author productivity analysis shows a clearly concentrated distribution, with a small core of researchers accumulating a significant volume of publications on *D. immitis*. In the 1945–2025 period, the most productive author was McCall J. (92 documents), followed by Morchón R. (84) and Carretón E. (72). Following them are Grieve R. (65), Simón F. (64), Christensen B. (60), Genchi C. (59), Otranto D. (54), Rawlings C. (53), and Montoya-Alonso J. (52). This pattern suggests the existence of long-standing scientific careers and consolidated research groups that have continuously sustained production in the field. When considering fractionalized productivity (which adjusts each author’s contribution based on the number of co-authors), the relevance of the main core remains, with important nuances. Notable figures include Rawlings C. (25.1), Christensen B. (23.7), Atwell R. (20.8), Grieve R. (19.9), Hayasaki M. (19.6), and McCall J. (19.3), indicating particularly consistent participation in collaborative publications (Table 3).

### 3.4. Most Relevant Sources

The dissemination of research on *D. immitis* is concentrated in a limited set of journals acting as the field’s primary channels. In the 1945–2025 period, Veterinary Parasitology was the most productive source (274 articles), followed by Parasites & Vectors (228), American Journal of Veterinary Research (172), and Journal of Parasitology (156). At a secondary level are the Journal of the American Veterinary Medical Association (136) and Parasitology Research (105), while Experimental Parasitology (83), American Journal of Tropical Medicine and Hygiene (78), and Molecular and Biochemical Parasitology (69) maintain a sustained contribution. The top 10 is completed by the Journal of Medical Entomology (57). This pattern is compatible with the existence of a specialized editorial core—in veterinary parasitology, veterinary medicine.

### 3.5. Scientific Production by Country

Analysis of scientific production by country reveals a clearly asymmetric geographic distribution, with a limited number of nations concentrating a substantial part of published research on *D. immitis*. The United States stands as the primary contributor with 803 articles, representing approximately 30% of total production, positioning it as the central hub of research in this domain. It is followed by Italy (238 articles) and Spain (167 articles), along with the United Kingdom (139 articles) and Brazil (127 articles), forming a second tier of highly productive countries. This pattern highlights the historical leadership of North America and Western Europe, alongside a prominent presence of countries from the Mediterranean area and Latin America. Collaboration type analysis shows relevant differences between countries. The United States presents a predominance of domestic authorship publications (SCPs), with a relatively low proportion of international collaborations (MCP ratio ≈ 0.14), whereas countries such as Italy, France, Germany, Portugal, and Romania exhibit notably higher international collaboration ratios, indicating greater integration into transnational scientific networks. In contrast, countries like India and Turkey show predominantly national production with limited participation in multinational publications. In terms of impact, measured by total citations, the United States again occupies the first position (19,575 citations), followed by Italy (9493) and the United Kingdom (4984). However, when considering average citations per article, countries such as Italy, the United Kingdom, Switzerland, and Germany stand out with high values, suggesting high visibility and influence of their publications, regardless of total production volume.

### 3.6. Most Cited Articles

The analysis of citation metrics reveals a field anchored by a few structural references. Simón et al. [2] stands out as the most influential work (619 citations), serving as a benchmark for the biology, epidemiology, and zoonotic potential of *D. immitis*. High-impact cross-disciplinary studies, such as those by Foster et al. [20] and McCall et al. [21], have driven the understanding of molecular mechanisms and Wolbachia, while specialized veterinary journals have cemented applied knowledge on diagnosis and control (e.g., Shoop et al. [24] and Traversa et al. [28]). Recent citation trends indicate a rapid uptake of One Health-related findings. Notably, these influential works largely correspond to the field’s most productive authors (Table 2 and Table 3), such as Genchi, Otranto, and Simón, confirming a stable core of international scientific leadership.

### 3.7. Keyword Analysis and Thematic Trends

**Figure 3:**
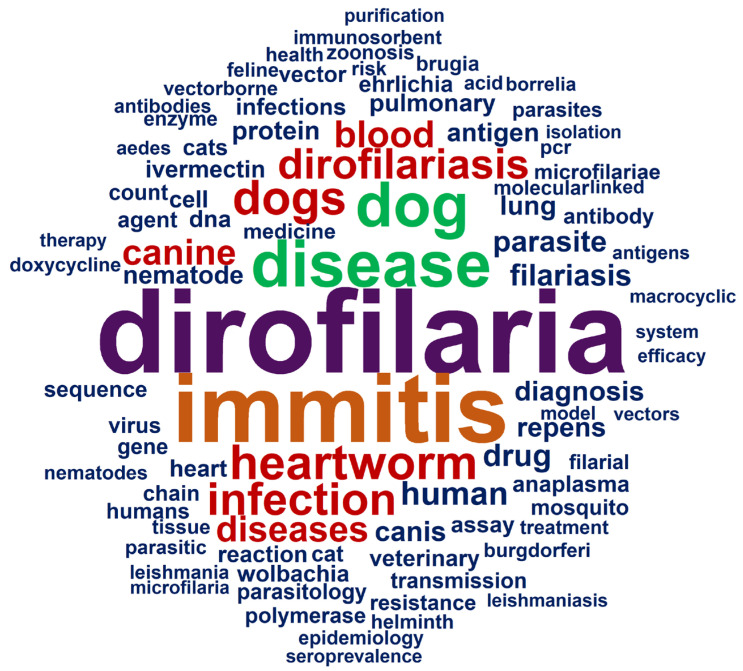
Keyword co-occurrence word cloud in *Dirofilaria immitis* research (1945–2025). The size of each term reflects its relative frequency across the analyzed corpus.

The word cloud generated from the corpus keywords visually and synthetically confirms the main thematic axes structuring *D. immitis* research during the analyzed period (Figure 3). The term “*Dirofilaria immitis*” appears clearly dominant, acting as the field’s conceptual core and articulating the remaining associated descriptors.

Grouped around this central term are keywords closely linked to the veterinary sphere, highlighting “dogs,” “dog,” “heartworm,” and “canine,” reinforcing the dog’s role as the definitive host and primary object of study. The recurrent presence of “dirofilariasis” and “heartworm disease” further underscores the consolidation of a well-defined clinical-epidemiological approach. Simultaneously, terms associated with epidemiology and disease burden, such as “prevalence,” “infection,” and “diagnosis,” acquire relevance, reflecting sustained interest in geographic distribution, risk factor identification, and diagnostic tool improvement. The prominent appearance of “animals,” “male,” and “female” also indicates the frequency of experimental and observational studies focused on host biological and population characteristics. Other relevant descriptors, such as “ivermectin,” “filariasis,” “nonhuman,” and “humans,” point to research lines related to treatment, prevention, and the disease’s zoonotic dimension. Although terms directly linked to human health present a lower relative weight, their presence confirms growing interest in the impact of *D. immitis* beyond the strictly veterinary realm, consistent with the One Health framework. Collectively, keyword analysis demonstrates that research on *D. immitis* is structured around three large, interrelated axes: (1) veterinary parasitology and clinical practice, (2) epidemiology and infection control, and (3) therapeutic and zoonotic aspects.

### 3.8. Evolution of Research Themes and Conceptual Structure (1945–2025)

**Figure 4:**
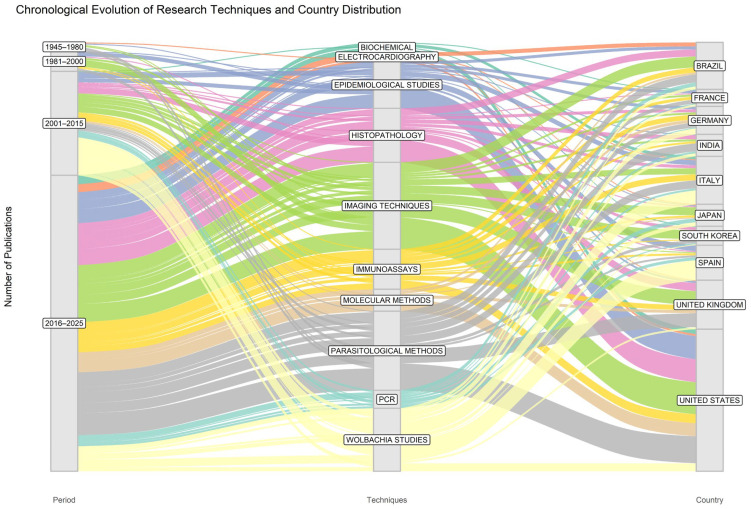
Evolution of research techniques and contributing countries in *Dirofilaria immitis* research across time periods with Sankey diagram. The diagram depicts the distribution and flow of publications across successive time periods, highlighting the diversification of methodological approaches and their association with major contributing countries.

The evolution of keywords and research themes reveals a progressive transformation of the field from initial descriptive studies to complex, integrative approaches (Figure 4). In the early decades (1970s–1980s), research was dominated by clinical and pathological descriptions (“congestive,” “etiology,” “in vitro study”), focusing on basic characterization. A shift occurred in the 1980s and 1990s toward experimental models and immunology (“animal disease,” “pulmonary artery,” “immunology”), consolidating the dog as the key host. By the turn of the millennium, the focus expanded to applied parasitology and diagnostics (“antigens,” “antibodies,” “disease transmission”). In the most recent period (2010–2025), there has been a decisive pivot toward One Health frameworks, with high-frequency terms such as “*Wolbachia*,” “zoonosis,” “molecular,” and “climate change” dominating the landscape. This trajectory confirms a maturation from single-discipline veterinary studies to a multidimensional scientific field.

**Figure 5:**
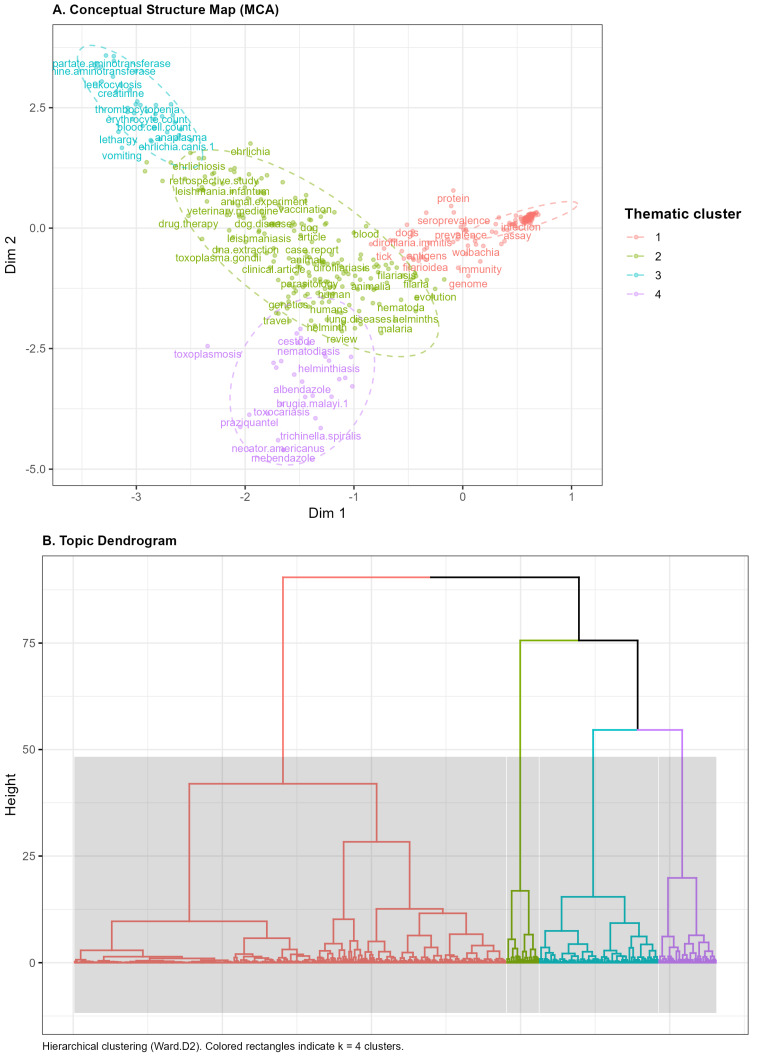
Conceptual structure of *Dirofilaria immitis* research derived from multiple correspondence analysis (MCA) of author keywords. (**A**) The conceptual structure map, where keywords are positioned according to their contributions to the first two MCA dimensions. (**B**) The hierarchical clustering dendrogram of keywords based on their MCA coordinates, illustrating the internal organization and relationships between thematic domains.

Structurally, this evolution is mirrored in the technical transition of the field. While early periods relied on traditional parasitological methods and imaging, the 2016–2025 era is characterized by the hegemony of molecular methods and advanced genomic studies. The Multiple Correspondence Analysis (MCA) and hierarchical clustering (Figure 5) validate this, identifying distinct thematic clusters ranging from classical clinical research to genomic control strategies. The conceptual structure map highlights that while highly cited papers tend to occupy a central, foundational position, recent peripheral documents are driving innovation through specialized molecular and zoonotic inquiries.

**Figure 6:**
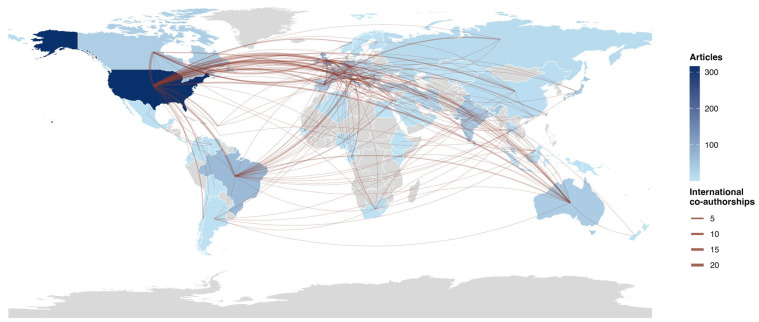
World map illustrating international scientific collaboration patterns in *Dirofilaria immitis* research based on co-authorship links between countries (1945–2025). Lines represent collaborative relationships between author-affiliated countries, while color intensity reflects the volume of scientific production.

Figure 6 presents the global collaboration map, highlighting the density of scientific cooperation networks. The results reveal a structure led by the United States and Italy as primary international hubs, linking research across the Americas, Europe, and Asia. Robust transatlantic collaboration is observed, alongside the emergence of key nodes in endemic regions such as Brazil and Australia.

The intellectual architecture of *D. immitis* research is structured into distinct domains. The MCA reveals strong cohesion between genomic studies and antiparasitic control (purple cluster), acting as the theoretical core. Conversely, clinical research (green and red clusters) shows technical specialization in symptomatology and differential diagnosis.

## 4. Discussion

The present study provides the first integral and longitudinal bibliometric analysis of global research on *D. immitis* over an 80-year period (1945–2025). The indicators obtained do not merely quantify scientific output but map the transformation of a discipline from classical veterinary parasitology toward a complex biomedical science. Collectively, these indicators describe a mature research field with sustained growth, consolidated scientific impact, and a predominantly collaborative authorship structure. The annual growth rate of 2.39% highlights the persistent relevance of this parasite, driven by its dual impact on small animal clinical practice and public health. It is worth noting that the slight reduction observed in the final years of the analyzed period should be interpreted with caution, as it may be influenced by indexing delays for the most recent documents rather than a genuine decline in scientific interest [29].

Regarding the geographical distribution of knowledge, the analysis reveals a strong polarization. The leadership of the United States and Italy, followed by Spain, correlates with the high prevalence of the parasite in these regions and the existence of long-standing schools of veterinary parasitology. However, a gap remains regarding highly endemic regions in the Global South. Collectively, these results demonstrate that research on *D. immitis* is dominated by a small group of countries with sustained, high-impact production, complemented by the more dispersed participation of other nations increasingly contributing to the field. This pattern is characteristic of consolidated research areas and reflects both structural inequalities in research capacity and distinct models of international scientific collaboration [6].

Despite these structural asymmetries, the network analysis indicates that leading countries do not work in isolation. There is a notable North–South flow of knowledge, where reference centers in Europe and North America collaborate with researchers in endemic areas. The predominant networks between developed nations and developing regions suggest a cooperative model aimed at mitigating vector expansion and improving control strategies across diverse climatic ecosystems, a crucial necessity given that vectors disregard political borders [7]. Overall, results reflect a structure typical of mature fields, with a small group of leading authors and a broad base of more dispersed contributions, consistent with the predominantly collaborative character of *D. immitis* literature.

From an intellectual perspective, the evolution of keywords and thematic clusters demonstrates a profound transformation. While early literature focused on morphology and acute clinical cases, the 21st century has introduced a molecular revolution. The most field-shaping contribution was the incorporation of *Wolbachia* into the conceptual and therapeutic framework of *D. immitis* research, as it shifted the field from descriptive parasitology toward molecularly informed control strategies [21,29]. This configuration demonstrates the field’s evolution from classical parasitology toward a multidisciplinary discipline integrating genomics with advanced clinical pathology. However, the factorial maps show that the basic science has not displaced clinical practice; rather, they have merged. This configuration demonstrates that *D. immitis* research has evolved into a diverse, multidisciplinary ecosystem where established clinical knowledge coexists with emerging genomic and environmental control strategies.

Furthermore, a pivotal factor reshaping the research landscape in the last decade has been the emergence and confirmation of macrocyclic lactone (ML) resistance in *D. immitis* populations. This biological crisis has acted as a catalyst for a surge in specialized studies focused on the genetic mechanisms of resistance, the search for single nucleotide polymorphism (SNP) markers, and the validation of alternative therapeutic protocols. The bibliometric increase in terms associated with “molecular,” “genetics,” and “efficacy” is largely driven by the urgent need to preserve the utility of current preventatives. This shift underscores that the field is not merely expanding, but actively responding to evolutionary pressures placed on the parasite by decades of chemical prophylaxis [25,30,31,32].

Additionally, the analysis highlights a growing intersection between veterinary parasitology and wildlife ecology. The persistence of dirofilariasis despite widespread preventive availability is increasingly attributed to the role of wild canids—such as coyotes in North America and golden jackals in Europe—as uncontrollable reservoirs. Recent literature reflects a concerted effort to map the overlapping interfaces between sylvatic and domestic cycles, acknowledging that the eradication of *D. immitis* is biologically implausible without addressing wildlife vectors. This realization has broadened the scope of epidemiological studies, shifting from simple prevalence surveys to complex ecological modeling [33,34].

Finally, the alignment of recent research with the One Health paradigm is evident in the keyword trends of the last decade. The focus has shifted toward zoonotic potential and the influence of climate change on transmission. The observed thematic coherence and stability of these descriptors over time reflect a mature field with well-defined research lines that continues to expand toward more integrative approaches connecting animal and human health [2]. While this study is limited by the exclusive use of indexed databases (WoS and Scopus), which may underrepresent regional literature, the consistency of the findings with previous systematic reviews validates the robustness of these global trends. It is also true that there are different issues that have not been addressed and that would be very interesting for the interpretation of the results, such as the underrepresentation of endemic regions with low research capacity, the lack of integration between epidemiological surveillance and climate modeling, the need for translational studies on the new resistances that have begun to appear especially in the American continent, the scarcity of studies that connect domestic and wild cycles, and the limited integration of the human zoonotic dimension versus the dominant veterinary weight.

## 5. Conclusions

This bibliometric study demonstrates that global research on *D. immitis* has evolved from a classical parasitology approach, focused on morphological and clinical description, into a multidisciplinary ecosystem aligned with the One Health paradigm. The identified intellectual structure reflects a balance between conceptual stability and adaptive capacity, allowing for the integration of methodological advances, new diagnostic tools, and emerging molecular approaches—such as *Wolbachia* targeting—while maintaining a solid foundation of clinical knowledge. Furthermore, the results highlight that, although structural inequalities persist in scientific production led by traditional research powers, there is a growing trend toward internationalization and collaborative work. In the current scenario of climate change and vector expansion, future research is prioritized toward integrative models that connect animal, human, and environmental health, transcending geographic and disciplinary boundaries to address dirofilariasis as a global health challenge.

## Figures and Tables

**Table 1:** The main information related to scientific production on *Dirofilaria immitis* (1945–2025).

Description	Results
Timespan	1945–2025
Sources (Journals, Books, etc.)	628
Documents	3589
Annual Growth Rate (%)	2.39
Document Average Age (Years)	19.0
Average Citations per Document	18.52
Total References	45,471
Author’s Keywords	4394
Authors	12,119
Authors of single-authored documents	215
Co-Authors per Document	5.41
International Collaboration (%)	16.58
Original articles	2638
Reviews	218
Other document types	733

**Table 2:** Most cited documents on *Dirofilaria immitis* (1945–2025). TC: Total citations; TCpY: Total citations per year; NTC: Normalized total citations.

Author, Year, Journal	DOI	TC	TCpY	NTC
Simón et al. [2]	10.1128/CMR.00012-12	619	41.27	16.26
Foster et al. [20]	10.1371/journal.pbio.0030121	493	22.41	15.42
McCall et al. [21]	10.1016/S0065-308X(08)00204-2	491	25.84	16.72
Bandi et al. [22]	10.1098/rspb.1998.0591	453	15.62	15.12
Fèvre et al. [23]	10.1016/j.tim.2006.01.004	384	18.29	11.09
Shoop et al. [24]	10.1016/0304-4017(94)00743-V	383	11.97	15.64
Wołstenhołme et al. [25]	10.1017/S003118201500061X	365	16.59	11.41
Beerntsen et al. [26]	10.1128/MMBR.64.1.115-137.2000	317	11.74	10.61
Rishniw et al. [27]	10.1016/j.vetpar.2005.10.013	256	12.19	7.39
Traversa et al. [28]	10.1186/1756-3305-3-62	247	14.53	12.58

**Table 3:** Top 10 most influential authors in *Dirofilaria immitis* research (1945–2025).

Author	h-Index	Scholarly Output	Citation Count	Citations/ Publication	Affiliation	Country
Genchi C	32	65	4464	68.7	University of Milan	Italy
Otranto D	31	86	3393	39.4	University of Bari	Italy
Morchon R	24	88	2305	26.2	University of Salamanca	Spain
Simon F	24	60	1954	32.6	University of Salamanca	Spain
Dantas-Torres F	24	40	2315	57.9	Fundação Oswaldo Cruz	Brazil
Mccall J	22	60	1902	31.7	University of Georgia	USA
Christensen B	22	53	1767	33.3	University of Wisconsin-Madison	USA
Carreton E	21	74	1794	24.2	University of Las Palmas de Gran Canaria	Spain
Chandrashekar R	18	37	922	24.9	IDEXX Laboratories	USA
Montoya-Alonso JA	15	49	591	12.1	University of Las Palmas de Gran Canaria	Spain

## Data Availability

Data are contained within the article or Supplementary Material.

## Supplementary Materials

The following supporting information can be downloaded at https://www.mdpi.com/article/10.3390/ani16060988/s1, Table S1: Compliance with PRISMA guidelines adapted for bibliometrics recommendations in the present bibliometric study.
