# Supplementary material for: Mapping Global Trends in Dirofilaria immitis Research Within the One Health Framework (1945–2025): A Bibliometric Perspective
Source: Animals (Basel). 2026 Mar 22;16(6):988. doi: 10.3390/ani16060988 (PMC13023904; doi:10.3390/ani16060988)
Supplement: Supplementary file 1 [file animals-16-00988-s001.zip › animals-4200410-supplementary.pdf]

Table S1. Compliance with PRISMA guidelines adapted for bibliometrics recommendations in the present bibliometric study.

RAMIBS item	Recommendation	Compliance	Section / Description
1. Title and abstract	Explanatory title reflecting the bibliometric approach; structured abstract	**Yes**	Title: 'Global research trends on Dirofilaria immitis within a One Health framework', explicitly identifying the subject and bibliometric approach. Structured abstract includes objectives, databases (WoS/Scopus), period (1945–2025), and key findings.
2. Introduction	Antecedents, relevance, research question, justification, and objectives	**Yes**	The introduction contextualizes Dirofilaria immitis within parasitology and the One Health framework, identifies knowledge gaps, and explicitly defines research justification and objectives.
3. Databases	Justification of database selection; inclusion/exclusion criteria; time period; deduplication	**Yes**	Web of Science Core Collection and Scopus were selected for their multidisciplinary coverage. Inclusion/exclusion criteria, time period, and deduplication procedures are described in the Methods section.
4. Search strategy	Detailed construction of search strategy, Boolean operators, and field selection	**Yes**	The search strategy was built using the scientific term 'Dirofilaria immitis', applied consistently across databases using Boolean operators in title, abstract, and keyword fields.
5. Search filters	Exact search date, document types, language restrictions	**Yes**	The analysis period, specific search date (January 2025), document types, and absence of language restrictions are explicitly detailed in the methodology to ensure reproducibility.
6. Bibliometric indicators	Use of core bibliometric dimensions (production, impact, collaboration, thematic)	**Yes**	Indicators of scientific production, impact (citations), collaboration (co-authorship/international), and thematic structure (keywords/evolution) were analyzed using the bibliometrix package.
7. Unit of analysis	Macro (countries), meso (institutions/topics), micro (authors)	**Yes**	Analysis was performed at macro (countries), meso (journals/themes), and micro (authors) levels, allowing a multi-scale interpretation of the scientific field.
8. Data extraction and processing	Deduplication, normalization, analytical techniques, justification	**Yes**	Records from both databases were merged. Deduplication was performed via DOI matching and title normalization, supplemented by manual review. Analysis was conducted using bibliometrix in R.
9. Presentation of data	Software versions, visualization parameters, normalization procedures	**Yes**	Data visualization and presentation were performed via bibliometrix (R environment), using standardized parameters for collaboration networks and thematic evolution analysis.
10. Results	Transparent presentation, interpretation, excluded records	**Yes**	Results are clearly presented through tables and figures with corresponding interpretation. A detailed flow diagram of included/excluded records is provided to ensure full transparency.
11. Discussion	Interpretation, comparison with other bibliometric studies,	**Yes**	Results are discussed in relation to previous bibliometric studies and current literature, highlighting major

RAMIBS item	Recommendation	Compliance	Section / Description
	limitations		implications and acknowledging inherent limitations.
12. Conclusion	Summary of main findings	**Yes**	The conclusion synthesizes the identified bibliometric patterns, global research trends on Dirofilaria immitis, and emerging lines within the One Health framework.
